# Impact of Highly Saturated versus Unsaturated Fat Intake on Carbohydrate Metabolism and Vascular Reactivity in Rat

**DOI:** 10.1155/2022/8753356

**Published:** 2022-08-19

**Authors:** Youzan Ferdinand Djohan, Fabrice Raynaud, Karen Lambert, Jean-Paul Cristol, Charles Coudray, Christine Feillet-Coudray, Anne Virsolvy, Eric Badia

**Affiliations:** ^1^Laboratoire de Biochimie, CHU, Université Félix Houphouët-Boigny, Cocody, Côte d'Ivoire; ^2^PhyMedExp, Université Montpellier, INSERM, CNRS, Montpellier, France; ^3^Laboratoire de Biochimie, CHU-Lapeyronie, Montpellier, France; ^4^DMEM, INRA, Université Montpellier, Montpellier, France

## Abstract

Palm olein (PO) and lard are considered harmful to health because of their highly saturated fatty acid content. On the contrary, olive oil (OO) with its high level of polyunsaturated fatty acids is considered healthier. This study aims to evaluate the effects of high consumption of these oils on carbohydrate metabolism and vascular function. Male Wistar rats were fed ad libitum for 12 weeks with different high fat diets (HFD) containing 30% of each oil. Systemic glycemia, insulinemia, and lipidemia were assessed by routine methods or by ELISA. GLUT4 muscular expression and hepatic and muscular Akt phosphorylation were analyzed by western blot. Vascular function was evaluated, ex vivo, on aortic rings and on the variations of isometric tensions. The results show that fasting blood glucose was increased with PO and OO diets and decreased with lard. Compared to control diet, this increase was significant only with PO diet. The area under the curve of IPGTT was increased in all HFD groups. Compared to control diet, this increase was significant only with PO. In contrast, stimulation of the pathway with insulin showed a significant decrease in Akt phosphorylation in all HFD compared to control diet. KCl and phenylephrine induced strong, dose-dependent vasoconstriction of rat aortas in all groups, but KCl EC_50_ values were increased with lard and OO diets. The inhibitory effect of tempol was absent in PO and lard and attenuated in OO. Vascular insulin sensitivity was decreased in all HFD groups. This decreased sensitivity of insulin was more important with PO and lard when compared to OO diet. In conclusion, the results of this study clearly show that high consumption of palm olein, olive oil, and lard can compromise glucose tolerance and thus insulin sensitivity. Furthermore, palm olein and lard have a more deleterious effect than olive oil on the contractile function of the aorta. Excessive consumption of saturated or unsaturated fatty acids is harmful to health, regardless of their vegetable or animal origin.

## 1. Introduction

A high-fat diet (HFD) over time promotes metabolic syndrome. The metabolic syndrome consists of many health problems, such as glucose intolerance, obesity, dyslipidemia, insulin resistance, and high blood pressure [1–4]. All these disturbances observed in metabolic syndrome increase the risk of the occurrence of cardiovascular diseases and type 2 diabetes [5]. Saturated fatty acids (SFA) are thought to be involved in the development of type 2 diabetes [6–8] and cardiovascular diseases [9, 10]. Monounsaturated fatty acids (MUFA) or polyunsaturated fatty acids (PUFA) are said to be beneficial to health [11–13]. The general consensus is that the dietary intake of SFA increases cardiovascular risk but that MUFA and PUFA intake decreases risk [10].

Cardiovascular diseases are caused by numerous changes in the arterial vasculature such as arterial stiffening [14], arterial wall thickening, and endothelial dysfunction [15]. To maintain the integrity of the artery in the presence of stimuli, the components of the arterial wall reorganize with the risk of increasing the potential for vascular dysfunction [16].

Palm olein (PO) and lard are rich in saturated fatty acids with similar compositions. Palm olein is composed of 50% SFA, 40% MUFA, and 10% PUFA, [17]. Lard is composed of 41% SFA, 47% MUFA, and 12% PUFA [18]. Olive oil (OO) is composed of 14% SFA, 77% MUFA, and 9% PUFA [19]. OO contains less saturated fatty acids and more monounsaturated fatty acids than PO or lard. The latter are considered to be harmful to health because of their composition in SFA. They also contain fewer compounds (vitamin E for PO and gallate for lard) with antioxidant properties [20, 21] known to attenuate the deleterious effect of SFA.

The aim of the present study was to evaluate the effects of long-term consumption of a hypercaloric diet high in saturated fatty acids versus unsaturated fatty acids on carbohydrate metabolism and arterial vessels' function. Thus, we compared the effects of HFD rich either in palm olein, lard, or olive oil on insulin resistance and vascular reactivity in rats.

## 2. Materials and Methods

### 2.1. Animals and Diets

The experiments complied with the guidelines for the care and use of laboratory animals (National Academies Press US, 8^th^ edition, 2011), and all procedures were approved by the local ethical committee (reference CEEA-LR-12002, Montpellier, France). Thirty-two young male Wistar rats aged 6 weeks coming from the Animal facilities of Charles River Laboratories, France, were used. The animals were housed under conditions of constant temperature (20–22°C), humidity (45–50%), and a standard dark cycle (20.00-08.00 h) with free access to food and water. Rats were randomly assigned to four groups of eight animals and fed for 12 weeks either a standard rat chow diet (control) or one of the three HFD. In the control diet, 11% of the energy was given by fat (5% soybean oil), whereas in enriched diets, 56% of the energy was provided by fat intake [22]. The fat-enriched diets consisted in 2.5% (w/w) of soybean oil and 30% (w/w) of PO (Sania Cie, Abidjan, Côte d'Ivoire), OO (virgin olive oil, bought in a supermarket), or lard (Alva, Rezé, France). The detailed composition of these experimental diets is given in Table 1.

### 2.2. Intraperitoneal Glucose Tolerance Test

The intraperitoneal glucose tolerance test (IPGTT) was completed seven days before rat sacrifice and performed as previously described [23, 24]. Briefly, after being fasting for 16 h, a glucose solution [2 g/kg body weight saline (0.9% NaCl)] was administered intraperitoneally. Blood was sampled through the tail vein of conscious rats immediately prior to the injection, and 20, 30, 40, 60, 90, and 120 min afterward. Blood glucose was measured using glucose strips and a commercial glucometer (AccuChek Active, Roche Diagnostics, USA). The area under the curve (AUC) was calculated by the trapezium method. The AUC values are expressed as *g* glucose per *L* per 120 min.

### 2.3. Rat Sacrifice and Sampling

Rats were fasted overnight, and blood was sampled via the abdominal artery under 1% pentobarbital anaesthesia (50 mg/kg ip). After centrifugation at 1000*g* for 10 min at 4°C, plasma was collected and stored at −80°C until analysis. Rat gastrocnemius was quickly removed; one piece was immediately frozen in liquid nitrogen and then kept at −80°C until analysis. The left soleus and right soleus were immersed, respectively, in a 0.7 *μ*M insulin solution or PBS without insulin at 37°C for 15 minutes. The two solei were then rinsed and frozen in liquid nitrogen.

### 2.4. Routine Biochemical Analyses

The plasma contents of glucose, total cholesterol, HDL cholesterol, and triglycerides, were determined using standard methods on a COBAS automated analyzer (Roche Diagnostics, France). Plasma insulin level was quantified by an immunoassay kit (Mercodia Rat Insulin ELISA). The homeostasis model assessment of insulin resistance (HOMA-IR) was calculated using the follow equation: HOMA-IR = [fasting blood glucose level (mmol·L^−1^) × fasting plasma insulin level (mUI·L^−1^)]/22.5.

### 2.5. Protein Isolation and Western Blot Analysis

Protein isolation and western blot analysis were performed as previously described [25]. Whole-cell protein lysates from gastrocnemius and soleus were prepared in the following lysis buffer: 20 mM Tris pH 8, 50 mM DL-dithiothreitol, 2 mM EDTA, 150 mM NaCl, 0.1% SDS, 1% Triton X100, 1 mM PMSF, 1 mM orthovanadate, and 1% (v/v) of antiprotease cocktail (Sigma-Aldrich, Saint-Quentin-Fallavier, France). Proteins were resolved by SDS-PAGE and then transferred to nitrocellulose membranes at 20 V overnight using refrigerated Tris-glycine transfer buffer. The membranes were blocked in 5% nonfat milk in PBS (without Tween) for 1 h at room temperature. Then, membranes were incubated overnight with primary antibody of protein kinase B (Akt), phospho-protein kinase B (p-Akt) (Cell Signaling, Leiden, The Netherlands); glucose transporter Glut 4 (GLUT 4) (Abcam, Cambridge, UK); tubulin (Sigma-Aldrich, Saint-Quentin-Fallavier, France) in blocking buffer. After washes in PBS/Tween under gentle agitation, the membranes were incubated for 45 min in the dark with the fluorescent-labeled secondary antibodies and finally quantified using the Odyssey infrared imaging system (LI-COR, Lincoln, USA).

### 2.6. Vascular Reactivity

The thoracic aorta was used to study *ex vivo* the responses to agonists and antagonists of arterial contraction as previously described [26]. Immediately after removal, arterial tissue was immersed in phosphate saline solution (PSS), pH 7.4, containing (in mM) 140 NaCl, 5 KCl, 1 MgCl_2_, 0.5 KH_2_PO_4_, 0.5 Na_2_HPO_4_, 2.5 CaCl_2_, 10 HEPES, and 10 glucose. Aortic tissue was cleaned of fat and connective tissue and cut into 2-3 mm wide rings. Aortic rings were mounted in standard organ bath chambers (EMKA Technologies, Paris, France) maintained at 37°C and continuously bubbled with O_2_. Then, the changes in isometric tension were recorded according to Fort et al. [27]. Each arterial segment was subjected to a 60 min equilibration period at the predetermined optimal basal tension of 2 g. The contractile function of each segment was assessed with 1 *µ*M phenylephrine (Phe), and the presence of endothelium was confirmed by the vasorelaxation induced by application of acetylcholine (Ach, 1 *µ*M). After several washouts and a 20–30 min period of stabilization, dose responses were performed by cumulative increases in the concentration of the agonist Phe (0.01–100 *µ*M range) or the depolarizing agent KCl (1–80 mM). Endothelial function was assessed by studying the relaxing effects of cumulative increases of Ach between 1 nM and 10 *µ*M on arteries contracted with a submaximally active concentration of Phe (10 *µ*M). The effects of the nitric oxide (NO) synthase inhibitor nitro-L-arginine methyl ester (L-NAME, 10 *µ*M) and the reactive oxygen species scavenger tempol (100 *µ*M) were evaluated on the contractile response to Phe and the relaxing effect of Ach. Inhibitors were added for a 15 min period of incubation before Phe addition. Endothelium-independent relaxations to sodium nitroprusside (SNP, 1 nM–200 *µ*M) were studied in endothelium-denuded rings previously contracted with Phe (10 *µ*M). Each protocol was performed in triplicate for each aorta. All chemical compounds were purchased from Sigma-Aldrich (Saint Quentin Fallavier, France).

### 2.7. Statistical Analysis

Values were expressed as mean ± SD, *n* = 7-8 animals per group. Statistical analysis was based on one-way ANOVA followed by a Tukey Kramer multiple comparisons test. When statistical variances were unequal, a Welch test was performed. Dose-response data were analyzed and compared by nonlinear regression methods using the PRISM software (GraphPad, San Diego, CA). Differences between dose–response curves were investigated by the half maximum effective concentration or sensitivity (EC50) and maximum effect (*E*_max_). The limit of statistical significance was set at *p* < 0.05. The group mean values with different letters (*a*, *b*, *c*, *d*) are significantly different. Statistical analyses were performed using the Stat-View program (SAS Institute, Cary, NC, USA).

## 3. Results

### 3.1. Effect of Diets on Body Weight, Lipid Parameters, and Antioxidant Defenses

As shown in Table 2, body weight was significantly increased (*p*=0.0083) by HFD (+15%) whatever the type of fat and with no difference between diets and type of fat. No increase in plasma total cholesterol and triglyceride levels was observed. Even in OO and lard groups, plasma levels of total cholesterol (*p*=0.0301) and HDL cholesterol (*p*=0.0106) were decreased. Compared to control diet, plasma HDL cholesterol was decreased with OO (−10.54%) and lard (−28.88%) diets and slightly increased with PO diet (+4.49%). Only lard significantly decreased HDL cholesterol compared to control diet. Blood SOD activity was decreased with PO and lard diets when compared to control and OO diets (Table 2).

### 3.2. Effect of Diets on Glucose Tolerance and Insulin Resistance

Fasting blood glucose was increased with PO (+13%) and OO (+8%) diets and decreased with lard (−5.43%). When compared to control diet, this increase was significant with PO (*p*=0.009) but not OO diet. With lard diet, glycemia was unchanged. Although no diet significantly changed fasting insulin levels and HOMA-IR, all HFD increased the value of these two parameters compared to control diet. HOMA-IR index was, however, higher with PO diet (Table 2).

**Figure 1:**
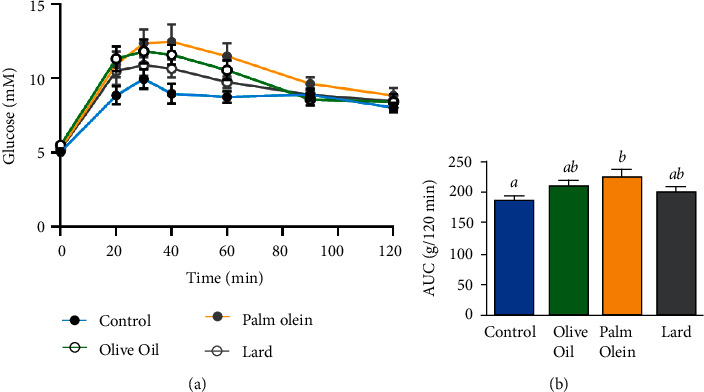
Blood glucose level evolution during intraperitoneal glucose tolerance test performed on 16 h-fasted animals (a) and calculated area under curve from IPTG (b). Results were expressed as mean values ± SD, *n* = 7-8 animals per group. The limit of statistical significance was set at *p* < 0.05. The group mean values with different letters (a, b, c) are significantly different. AUC: area under the curve; IPGTT: intraperitoneal glucose tolerance test.

Whole body glucose tolerance was investigated with intraperitoneal glucose injection (IPTG) (Figure 1). No significant differences were observed between groups at T0, T20, T30, T90, and T120 minutes. However, compared to the control diet, with OO and PO diets, blood glucose at T40 (*p*=0.0223) and T60 minutes (*p*=0.0189) was significantly higher (Figure 1(a)). The area under the curve (AUC) of IPGTT was increased in all HFD groups (PO: +20.96%, OO: +13.44%, lard: +8.60%). Compared to control diet, this increase was significant only with PO (*p*=0.0263), and no difference was observed between the different HFD (Figure 1(b)). These results evidenced a decrease in glucose handling with diets and especially PO.

**Figure 2:**
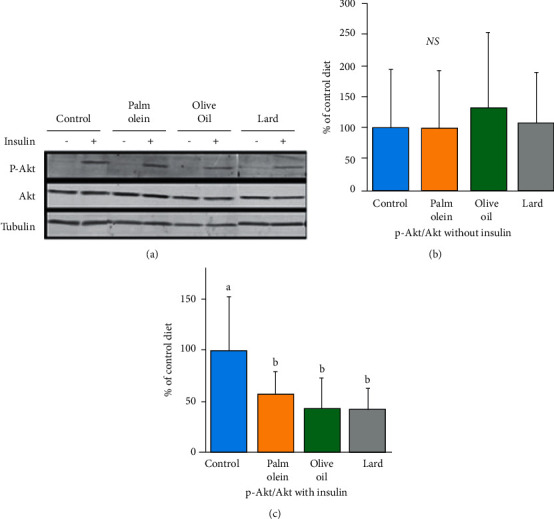
Effects of diets on phospho-Akt protein expression in muscle (soleus) in the absence or presence of insulin. Representative western blot of either total Akt or phospho-Akt with tubulin as a normalizing gene (a). The histograms of blot quantification in the absence of insulin (b). The histograms of blot quantification in the presence of insulin (c). Results were expressed as mean values ± SD, *n* = 7-8 animals per group. The limit of statistical significance was set at *p* < 0.05. The group mean values with different letters (a, b, c) are significantly different. Akt or PKB: protein kinase B; p-Akt: phosphorylated Akt; NS: not significant.

Since skeletal muscles are major regulators of insulin-stimulated glucose uptake, we investigated muscle insulin resistance with the p-Akt/Akt ratio. Insulin signaling pathway was evaluated in soleus by the level of Akt phosphorylation. No significant difference was observed between groups in basal Akt activation as assessed by the p-Akt/Akt ratio (Figure 2). In contrast, stimulation of the pathway with insulin showed a significant decrease (*p*=0.0057) in Akt phosphorylation in all HFD compared to control diet. However, no differences between HFD themselves were found (Figure 2). Together with the trend toward increased insulin level and HOMA-IR in HFD groups, these results are in favor of a decreased insulin sensitivity at the whole body and muscle level. Muscle expression of the glucose transporter GLUT4 was not affected by the diets (Supplemental Figure 1).

### 3.3. Effect of Diets on Vascular Function

**Figure 3:**
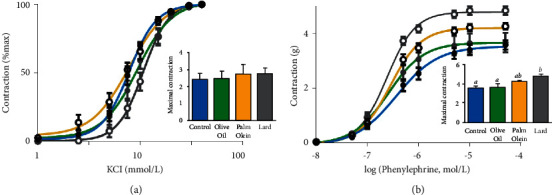
Contractile responses of rat aorta. Graphs represent cumulative response curves (a) to KCl (1–80 mM) and (b) to phenylephrine (0.01–100 *μ*M). Histograms represent the maximal contraction (*E*_max_) induced by a maximally active concentration of KCl (a) and phenylephrine (b). Results were expressed as mean values ± SD, *n* = 7-8 animals per group. The limit of statistical significance was set at *p* < 0.05. The group mean values with different letters (a, b, c) are significantly different. KCl: potassium chloride.

Contractile properties were evaluated on aortic rings with the depolarizing agent KCl and with the *α*-adrenergic agonist phenylephrine (Phe). Both agonists induced strong, dose-dependent vasoconstriction of rat aortas in all groups (Figure 3). Concerning KCl-induced responses, no significant difference was observed between groups on Emax values (Figure 3(a)), but EC50 values were increased with lard (+32%) and OO (+13%) diets. This increase was significant (*p*=0.03) with lard diet compared to control diet (Table 3). This change, reflected by a rightward shift of the dose-response curves, traduced an increased sensitivity to depolarization consecutive to aortic tissue hyperpolarization.

The contractile responses to Phe (0.01–100 *μ*M) were increased in the aortas of rats fed with PO and lard, while no difference was observed in the OO group compared to the control group (Figure 3(b)). EC50 values, illustrating Phe sensitivity, were not modified by the diets (Table 3).

**Figure 4:**
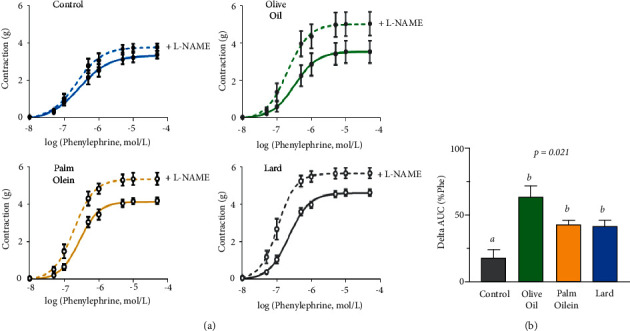
Effect of L-NAME on the contractile response to Phe. (a) Concentration-response curves to phenylephrine (0.01–100 *µ*M) in aortic rings from control, olive oil, palm olein, and lard diets in absence and in presence of L-NAME (10 *µ*mol/L). (b) Histogram represents the difference in area under curve between contractions with and without L-NAME for each group expressed as percentage of Phe effect. Results were expressed as mean values ± SD, *n* = 7-8 animals per group. The limit of statistical significance was set at *p* < 0.05. Histogram bars with different letters (*a*, *b*, *c*) are significantly different. AUC: area under the curve; L-NAME: nitro-L-arginine methyl ester.

**Figure 5:**
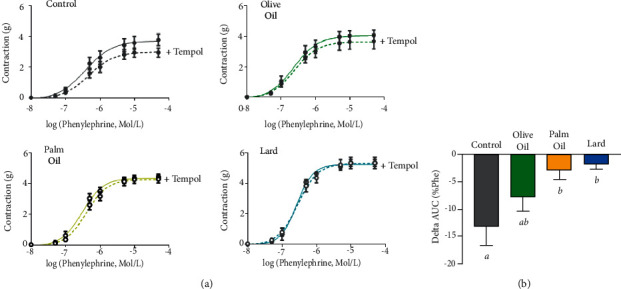
Effect of tempol on the contractile response to Phe. (a) Concentration–response curves to phenylephrine (0.01–100 *µ*M) in aortic rings from control, olive oil, palm olein and lard diets in absence and in presence of tempol (100 *µ*mol/L). (b) Histogram represents the difference in area under curve between contractions with and without tempol for each group expressed as percentage of Phe effect. Results were expressed as mean values ± SD, *n* = 7-8 animals per group. The limit of statistical significance was set at *p* < 0.05. Histogram bars with different letters (*a*, *b*, *c*) are significantly different. AUC: area under the curve.

To determine whether contractile response alterations were due to changes in basal NO contribution, we analyzed the differences in Phe response curves performed in the presence of L-NAME (Figure 4) and of tempol (Figure 5). Preincubation with L-NAME, a nitrate synthase inhibitor, enhanced contractile responses to Phe in all groups. This increase is due to inhibition of NO production and suppression of its subsequent vasorelaxant tone. This effect was markedly more important in HFD groups (*p*=0.021) and especially with OO diet when compared to control group (Figure 4(a)) as depicted by area under curve analysis (Figure 4(b)).

Conversely, incubation with tempol, a SOD mimetic and ROS scavenger, decreased the contractile response to Phe (Figure 5(a)) due to O_2_^−^ dismutation and suppression of O_2_^−^-induced contractile tone. This inhibitory effect of tempol was absent in PO and lard and attenuated in OO as depicted by AUC analysis (Figure 5(b)).

**Figure 6:**
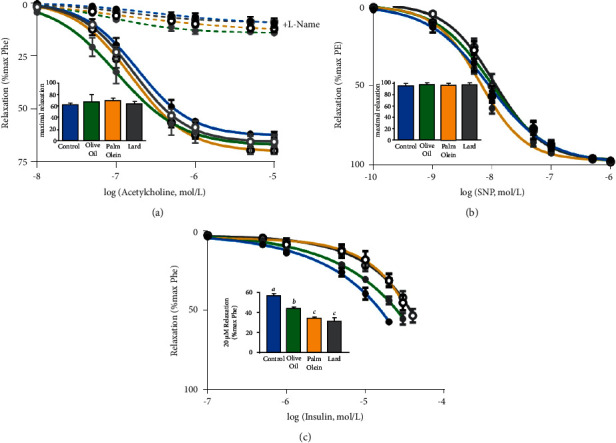
Effects of diets on relaxation of phenylephrine-precontracted aortic rings. Endothelium-dependent relaxation induced by acetylcholine (a) and endothelium-independent relaxation induced by SNP (b) and relaxant effect of insulin (c). Graphs represent cumulative response curves to acetylcholine (0.01–10 *µ*M) in the absence and in the presence of L-NAME (10 *µ*M), SNP (0.1 nM–1 *µ*M) and Insulin (0.1–50 *µ*M) on aortic rings previously contracted with 10 *µ*M Phe. Histograms represent the maximal relaxation obtained for Ach and SNP and the relaxation induced by 20 *µ*M insulin. Results were expressed as mean values ± SD, *n* = 7-8 animals per group. The limit of statistical significance was set at *p* < 0.05. The group mean values with different letters (*a*, *b*, *c*) are significantly different. Ach: acetylcholine; L-NAME: nitro-L-arginine methyl ester; SNP: sodium nitroprusside.

Relaxant properties were evaluated with the antagonists Ach and SNP to, respectively, investigate endothelium-dependent and endothelium-independent relaxation. Ach-induced dose-dependent relaxation of previously contracted arterial rings reflected endothelial function (Figure 6(a)). SNP, a NO donor, also induced dose-dependent relaxation of arterial rings that involved smooth muscle cells (Figure 6(b)). In the different groups, we observed no modification of either endothelial function based on acetylcholine-induced endothelium-dependent relaxation (0.01–100 *µ*M) (Figure 6(a)) or SNP-induced endothelium-independent relaxation (0.01–200 *µ*M) (Figure 6(b)). No significant difference between control and OO, PO, and lard diets either for the *E*_max_ and the EC_50_ values for both Ach and SNP was detected (Figure 6 and Table 3).

In the presence of L-NAME, Ach-induced relaxation was strongly inhibited (Figure 6(a)) in all groups. No difference was observed between groups for L-NAME effect. In the presence of tempol, Ach-induced relaxation was slightly increased in all groups with no difference observed between groups (not shown).

As insulin resistance is characterized by the inability of insulin to induce proper signal transduction leading to impaired insulin-induced vasodilation, we studied the vasorelaxant properties of insulin on Phe-contracted aortic rings. We observed that insulin induced a dose-dependent vasorelaxant response in aortic rings for all groups (Figure 6(c)). Vascular insulin sensitivity was decreased in all HFD groups when compared to control as shown by the relaxant response to 20 *µ*M insulin (bar graph in Figure 6(c)). This decreased sensitivity of insulin was more important with PO and lard when compared to OO diet.

## 4. Discussion

Although numerous studies have shown vascular dysfunction in rat on HFD, no studies to date have compared the effects of saturated fatty acids versus unsaturated fatty acids in diets. In the present study, we show that any excess of fat, whatever its origin (vegetable or animal) and its nature (saturated or unsaturated) induced weight gain is, disrupted lipidemic status and carbohydrate metabolism and decreased insulin sensitivity. However, only diets enriched with saturated fatty acids (PO and lard) increased arterial procontractile tone involving reduced NO bioavailability and increased oxidative stress.

The disruption of carbohydrate metabolism, highlighted by many authors [28–33], was marked by muscle insulin resistance induced by significant decrease in Akt activation [34, 35], which promoted the increase in insulin levels [36] and HOMA-IR with all HFD. Otherwise, it has been observed that high OO diet over a long period of time induces obesity and insulin resistance similar to what is observed with lard-based diet [37]. Our results showed that all HFD promoted insulin resistance, which was more important with PO. Decreased vascular insulin sensitivity was also observed with PO and with lard diets. In our model, PO seems more diabetogenic than lard and OO. Moreover, the lipid status is modified even if it is not strongly altered. HDL-C was reduced with lard and less significantly with OO. According to many authors [38, 39], the decrease of HDL-C is one of the major risk factors for the occurrence of cardiovascular diseases (CVD). The role of SFA in CVD is related to their carbon number and position on the triglycerides [40, 41]. In fact, cardiovascular risk factors (Total Cholesterol, LDL-C, HDL-C, and VLDL-C) are more important when the main SFA in the diet are short-chain FAs: lauric acid (C12:0) and myristic acid (C14:0) [41, 42]. The atherogenicity of a diet is related to the degree of saturation of the fatty acids located in position sn-2 of the triglycerides [43, 44]. In PO, 85% of the fatty acids in the sn-2 position are MUFA or PUFA [43, 45], whereas in lard, 61% of the fatty acids in the sn-2 position are SFAs, notably palmitic acid [46]. This arrangement of fatty acids on triglycerides could explain the normal profile induced by PO and the atherogenic profile induced by lard compared to control diet.

Many studies have shown that high fat diets contribute to vascular dysfunction. In animal models, abnormalities in vascular reactivity consist primarily of increased contractility and reduced vasodilation associated to endothelial dysfunction [47]. Endothelial function can be reflected by acetylcholine-induced relaxation [26]. In this study, we observed that none of the diets disrupted acetylcholine-induced relaxation, showing that PO, OO, and lard diets did not elicit endothelial dysfunction in our model. In contrast, PO and lard HFD promoted a contractile dysfunction revealed by an increase in the maximal Phe-induced contraction. The fact that the diets in the study did not promote endothelial dysfunction could be explained, on the one hand, by their antioxidant content. OO and PO contain several classes of minor components such as polyphenols, carotenoids, and vitamin E, which have antioxidant properties [48–50]. Lard on the market is enriched with gallate, which is a powerful antioxidant [51]. Indeed, many studies [52–54] have highlighted the role of antioxidants in improving endothelial function.

On the other hand, whether endothelial dysfunction is a common feature of HFD-induced vascular alterations, numerous studies report no difference in Ach-induced endothelium-dependent relaxation of arteries [55], and early endothelial dysfunction may be masked by compensatory mechanisms. Interestingly, it has been shown in some models that insulin-induced vasodilation, which is also endothelium-dependent, is impaired earlier than dilation to acetylcholine [56]. In our study, we evidenced decreased vascular insulin sensitivity with HFD and especially with PO and lard. Such mechanisms could occur in our model. Enhanced vasoconstriction, as a result of enhanced oxidative stress and ROS production, has been documented in insulin resistance [57].

The endothelium constitutively produces NO and hyperpolarizing factors, which participate in baseline tone through anticontractile activity [58]. NO signaling depends on a delicate balance between NO production via endothelial NO synthase and its inactivation by ROS such as superoxide [59]. In the presence of L-NAME, which is an inhibitor of NO synthase, the vasorelaxant tone of NO is suppressed, and the response to Phe is increased. We observed that the effect of L-NAME on Phe-induced response is potentiated in all HFD groups suggesting that NO or NO metabolites participation in baseline tone was increased. This could reflect an alteration in either NO production or signaling. As no endothelial dysfunction was evidenced in our model, altered NO production is ruled out in favor of impaired NO signaling and bioavailability. NO scavenging by superoxide anion (O_2_^−^) could be increased leading to increased peroxynitrite production and hypercontractility. Consistent with this hypothesis and with a previous study [60], on the one hand, we observed a decreased SOD activity with PO and lard diet. On the other hand, the effect of tempol on Phe-induced contractile response was significantly decreased with PO and lard diets, indicating that O_2_^−^ is not the main ROS involved in vascular tone.

Taken together, our results demonstrated that all HFD altered vascular function and NO availability rather than NO production. With PO and lard diets, a vascular procontractile dysfunction associated to increased oxidative stress and NO oxidation is observed, while, in comparison with OO diet, NO oxidation is reduced in favor O_2_^−^ dismutation.

In conclusion, the results of this study clearly show that high consumption of PO, OO, and lard can compromise glucose tolerance and thus insulin sensitivity. Furthermore, PO and lard have a more deleterious effect than OO on the contractile function of the aorta. Excessive consumption of saturated or unsaturated fatty acids is harmful to health, regardless of their vegetable or animal origin. As these results are not necessarily transposable to usual fat consumption, it would be interesting to study the effects of increasing doses of PO, OO, or lard to determine the adequate dose that does not compromise insulin sensitivity and aortic contractility. Further studies are needed to assess the effect of long-term consumption of OO, PO or lard in nonobesogenic diets in human.

## Figures and Tables

**Table 1:** Diet composition (g/kg) based on the AIN-93M diet formulation.

Ingredients	Control	Olive oil	Palm olein	Lard
Casein	165	200	200	200
Corn starch	443	234	234	234
Maltodextrin	144	80	80	80
Sucrose	100	53	53	53
Soybean oil	50	25	25	25
Olive oil	0	300	0	0
Palm olein	0	0	300	0
Lard	0	0	0	300
Cellulose	50	50	50	50
Mineral mix (AIN-93M)	35	42	42	42
Vitamin mix (AIN-93M)	10	12	12	12
L-cystine	2	2.4	2.4	2.4
Choline chloride	1.5	1.8	1.8	1.8
TOTAL	1000	1000	1000	1000

**Table 2:** Body weight and metabolic parameters in various groups.

	Control	Olive oil	Palm olein	Lard	*p*
Body weight (g)	519 ± 25^a^	588 ± 49^b^	605 ± 52^b^	611 ± 66^b^	0.0083
Total cholesterol (mmol/L)	2.00 ± 0.41^a^	1.72 ± 0.28^ab^	1.89 ± 0.46^a^	1.55 ± 0.26^b^	0.0301
HDL cholesterol (mmol/L)	1.49 ± 0.30^a^	1.33 ± 0.20^ab^	1.48 ± 0.36^a^	1.06 ± 0.33^b^	0.0106
Triglycerides (mmol/L)	1.18 ± 0.55	1.01 ± 0.31	1.21 ± 0.60	1.01 ± 0.42	NS
Glucose (mmol/L)	7.73 ± 0.24^a^	8.35 ± 0.35^ab^	8.73 ± 0.21^b^	7.31 ± 0.32^c^	0.009
Insulin (µg/L)	2.13 ± 0.98	3.37 ± 1.01	5.17 ± 1.24	3.73 ± 0.94	NS
HOMA-IR	17.3 ± 2.5^a^	31.6 ± 10.3^a^	55.1 ± 10.8^b^	30.3 ± 8.3^a^	0.023
SOD (U/mL)	324.2 ± 5.4^a^	326.7 ± 6.6^a^	262.1 ± 8.8^b^	275.9 ± 6.2^b^	0.0021

Results were expressed as mean values ± SD, *n* = 7-8 animals per group. The limit of statistical significance was set at *p* < 0.05. The group mean values with different letters (*a*, *b*, *c*) are significantly different. HDL: high density lipoprotein; HOMA-IR: Homeostasis model assessment of insulin resistance; SOD: superoxide dismutase; NS: not significant.

**Table 3:** Effects of diets on half maximum effective concentration (EC_50_) for agonists (Phe, KCl) and antagonists (Ach, SNP).

	Control	Olive oil	Palm olein	Lard	*p*
EC_50_ KCl (mM)	7.97 ± 0.5^a^	9.03 ± 0.7^ab^	7.63 ± 0.8^a^	10.58 ± 0.6^b^	0.03
EC_50_ phe (mM)	368 ± 41	254 ± 27	279 ± 30	240 ± 22	NS
EC_50_ ach (nM)	206 ± 50	157 ± 46	164 ± 32	166 ± 30	NS
EC_50_ SNP (nM)	6.5 ± 0.5	9.5 ± 0.8	6.9 ± 0.5	9.9 ± 1.2	NS

Results were expressed as mean values ± SD, *n* = 7–8 animals per group. The limit of statistical significance was set at *p* < 0.05. The group mean values with different letters (*a*, *b*, *c*) are significantly different. Ach: acetylcholine; KCl: potassium chloride, Phe: phenylephrine; SNP: sodium nitroprusside.

## Data Availability

The data used to support the findings of this study are included within the article.

## Abbreviations

Ach: Acetylcholine
AUC: Area under the curve
HFD: High-fat diet
HOMA-IR: Homeostasis model assessment of insulin resistance
IPGTT: Intraperitoneal glucose tolerance test
KCl: Potassium chloride
L-NAME: Nitro-L-arginine methyl ester
MUFA: Monounsaturated fatty acids
NO: Nitric oxide
OO: Olive oil
Phe: Phenylephrine
PO: Palm olein
PUFA: Polyunsaturated fatty acids
SFA: Saturated fatty acids
SNP: Sodium nitroprusside
SOD: Superoxide dismutase
NS: Not significant.

## Supplementary Materials

Supplementary Materials Supplemental Figure 1: Effects of diets on total GLUT4 protein expression in muscle Representative western blot of total GLUT4 with tubulin as a normalizing gene (a). The histograms of blot quantification (b). Results were expressed as mean values ± SD, *n* = 7-8 animals per group. The limit of statistical significance was set at *p* < 0.05. NS: not significant.
