# Supplementary material for: Impact of Highly Saturated versus Unsaturated Fat Intake on Carbohydrate Metabolism and Vascular Reactivity in Rat
Source: Biochem Res Int. 2022 Aug 19;2022:8753356. doi: 10.1155/2022/8753356 (PMC9417764; doi:10.1155/2022/8753356)
Supplement: Supplementary Materials — Supplemental Figure 1: Effects of diets on total GLUT4 protein expression in muscle Representative western blot of total GLUT4 with tubulin as a normalizing gene (a). The histograms of blot quantification (b). Results were expressed as mean values ± SD, n = 7-8 animals per group. The limit of statistical significance was set at p < 0.05. NS: not significant. [file 8753356.f1.docx]

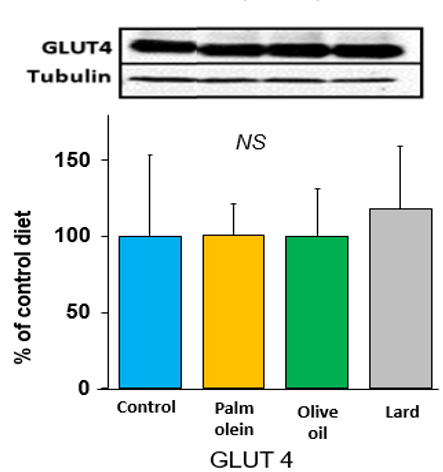


**A**

**B**

**Supplemental Figure 1:** Effects of diets on total GLUT4 protein expression in muscle

Representative western blot of total GLUT4 with tubulin as a normalizing gene (A). The histograms of blot quantification (B). Results were expressed as mean values ± SD, n = 7–8 animals per group. The limit of statistical significance was set at p < 0.05. NS: not significant
